# Erector spinae plane block versus thoracic paravertebral block in pediatric patients undergoing percutaneous nephrolithotomy: a prospective randomized clinical trial

**DOI:** 10.1016/j.bjane.2026.844735

**Published:** 2026-02-04

**Authors:** Fatma Nabil, Ahmed M. Mandour, Amr M. Abdelgawad, Deiaaeldin M. Tamer, Ahmed A. Shahat, Mohamed Anwar, Hany M. Osman

**Affiliations:** a Assiut University, Faculty of Medicine Department of Anesthesia Intensive Care and Pain Management, Assiut, Egypt; b Assiut University, Faculty of Medicine Department of Urology, Assiut, Egypt; c Assiut University, Faculty of Medicine Pediatric Department, Assiut, Egypt

**Keywords:** Analgesia, Anesthesia, Block, Pediatric, Percutaneous nephrolithotomy, Postoperative pain

## Abstract

**Background:**

Percutaneous Nephrolithotomy (PCNL) is a well-established surgical procedure for removal of large, multiple, and complex renal calculi in children. Combining loco-regional techniques with general anesthesia has gained increasing popularity in pediatric anesthesia. The objective of this trial was to evaluate the efficacy of Erector Spinae Plane Block (ESPB) versus thoracic Paravertebral Block (PVB) in pediatric patients undergoing PCNL procedure.

**Methods:**

Fifty-six children, aged 2‒7 years, who underwent PCNL procedure under general anesthesia were randomly assigned to receive either ESPB (n = 28) or thoracic PVB (n = 28) with the same anesthetic mixture of 0.3 mL.kg^-1^ bupivacaine 0.25% in epinephrine 1:100000. The primary outcome was time to first rescue analgesia (nalbuphine).

**Results:**

The time to first rescue analgesia was 15.98 ± 10.17 hours (95% CI: 12.14–19.82) in the ESPB group versus 18.18 ± 9.18 hours (95% CI: 14.7–21.58) in the thoracic PVB group with no significant difference (p = 0.464, log-rank test). Moreover, the total dose and frequency of administration of nalbuphine during the first 24 postoperative hours were comparable between the two studied groups (p = 0.488 and 0.479 respectively). However, the time to conduct the block was significantly shorter in the ESPB group versus the thoracic PVB group (4.37 ± 1.08 minutes vs. 5.05 ± 1.17 minutes respectively, p = 0.028).

**Conclusion:**

ESPB was not found to be more effective than thoracic PVB for postoperative pain management in children undergoing PCNL procedure. Moreover, intraoperative hemodynamics and maximum sevoflurane concentration were comparable. The time to conduct ESPB was significantly shorter; hence, it could be considered as an easy alternative to thoracic PVB for this procedure.

## Introduction

Over the past decades, Percutaneous Nephrolithotomy (PCNL) has become a well-established procedure for extraction of large, multiple, and complex renal stones in pediatric patients.^1^ Despite being a minimally invasive procedure, patients still experience postoperative pain that is attributed to renal capsule & parenchymal tract dilation, the creation of access tract via the tissues, and the nephrostomy tube inserted by the end of the procedure.^2^

Nowadays, regional blocks are frequently used as adjuvants to general anesthesia in pediatric patients to achieve better operating conditions and less opioid consumption, and to provide better postoperative analgesia.^3^

Paravertebral Block (PVB) has been used effectively for pain relief in pediatric patients after renal surgeries including PCNL procedure.^4^^,^^5^ Nevertheless, it requires high experience to minimize probable complications such as pneumothorax, pleural puncture, epidural or intrathecal spread, or vascular injury.

Erector Spinae Plane Block (ESPB) is a recently introduced interfascial plane block that was first conducted for neuropathic pain management.^6^ Thereafter, ESPB was reported for management of postoperative pain in numerous surgical procedures in pediatric patients.^7^, ^8^, ^9^

The objective of this trial was to test whether ESPB is more effective than thoracic PVB block for management of postoperative pain in pediatric patients undergoing PCNL procedure considering the time to first rescue analgesia as the primary outcome.

## Materials methods

### Trial design, setting, and ethics

This prospective, randomized, assessor-blind, superiority clinical study was performed at Assiut University Urology Hospital after approval of its protocol by the Institutional Review Board (IRB) of the Faculty of Medicine, Assiut University, Egypt, on July 21, 2022 (IRB number: 17300787). It was also registered in clinicaltrials.gov (NCT05589649) before recruitment. The study protocol was discussed with the parents/legal guardians of the participants prior to obtaining written informed consents. This study was carried out in accordance with the Helsinki declaration (2013) and considering the Consolidated Standards of Reporting Trials (CONSORT) guidelines, including the flow diagram.^10^

### Participants, randomization, and masking

The inclusion criteria were children aged 2 to 7 years with American Society of Anesthesiologists (ASA) physical status class I or II who were scheduled for PCNL procedure. Exclusion criteria were refusal of the parent or legal guardian, coagulation disorders, local site infection, known allergy to the used drugs, spinal cord abnormalities, or neurological deficits.

Based on a computer-generated random list conducted by a biostatistician, the children who fulfilled the inclusion criteria were allocated either to the ESPB group (n = 28) or thoracic PVB group (n = 28). Opaque, sealed envelopes were used to conceal the allocation and were unsealed in the morning of the procedure by a nurse who had no further roles. For all patients, the regional blocks were conducted by the same anesthesiologist who is well-experienced in ultrasound-guided regional blocks in pediatric patients. This anesthesiologist had no further participation in the perioperative management. Another anesthesiologist, who was unaware of the regional block performed, was responsible for intraoperative monitoring and management according to the study protocol. Furthermore, assessment and management of postoperative pain were achieved by a pediatrician and trained nurses who were unaware of the group allocation.

### General anesthesia and regional blocks

Thirty minutes before shifting to the Operative Theatre (OT), all children were sedated orally with midazolam (0.5 mg.kg^-1^, maximumly 15 mg). Inhalational induction of anesthesia was performed with sevoflurane (2%‒8%). Thereafter, propofol (1 mg.kg^-1^), cis-atracurium (0.15 mg.kg^-1^), and fentanyl (1 µg.kg^-1^) were administered to facilitate intubation then ventilation with parameters adjusted to keep EtCO_2_ as 35‒40 mmHg. Anesthesia was continued with sevoflurane (1‒2 MAC) and incremental doses of cis-atracurium.

After ensuring stable hemodynamic parameters, the patient was turned into prone position then under fully aseptic conditions, either ESPB or thoracic PVB was performed according to the patient’s group allocation. For all patients, blocks were given under ultrasonographic guidance using a linear transducer (Vivid S6, GE, 4‒13 MHz). For both blocks, the same volume was given as 0.3 mL.kg^-1^ of the anesthetic mixture (bupivacaine 0.25% in epinephrine 1:100000) in the ipsilateral side of the surgical procedure.

In the ESPB group: T11 spinous process was identified by counting from C7 spinous process downwards. The ultrasound transducer was then applied at this level in transverse position to locate the tip of the T11 transverse process. The transducer was then rotated to a longitudinal position to visualize the Erector Spinae Muscle (ESM). Thereafter, a 22-G echogenic needle (50–80 mm) was inserted using the in-plane technique in a caudal-to-cranial direction aiming to contact T11 transverse process. After negative aspiration, the accurate needle tip position was verified by injecting 1‒2 mL saline to elevate the ESM. Finally, the anesthetic mixture was injected.

In the thoracic PVB group, T10 and T11 spinous processes were identified by counting from C7 spinous process downwards. The ultrasound transducer was then applied in a parasagittal position slightly lateral to T10 and T11 spinous processes to visualize the superior costotransverse ligament, pleura, and paravertebral space. Thereafter, the needle was inserted using the in-plane technique in a caudal-to-cranial direction to penetrate the ligament targeting the T10‒T11 paravertebral space. After negative aspiration, hydrolocalization was done by injecting 1‒2 mL saline to confirm the correct position of the needle tip by displacing the ipsilateral pleura anteriorly. Finally, the same anesthetic mixture was injected.

Surgery was authorized to begin 15 minutes after the regional blockade. Rise of the Heart Rate (HR) > 20% in response to the surgical procedure was controlled first by increasing the inspired concentration of sevoflurane to 4% and then with a single bolus dose of fentanyl (1 µg.kg^-1^). Prior to the end of surgery, IV dexamethasone (0.2 mg.kg^-1^), ondansetron (0.1 mg.kg^-1^), and paracetamol (15 mg.kg^-1^) were administered to the patient. After returning the patient to the supine position; sevoflurane was turned off, and the neuromuscular agent was reversed. After extubation, the child was transferred to the Post-Anesthesia Care Unit (PACU) for two hours and then to a surgical intermediate care unit.

### Surgery

The surgical technique was standardized for all patients as follows: while the patient in lithotomy position, the urethra was anesthetized with lidocaine gel then, a 5-French ureteric catheter was inserted. The patient was then put into prone position with careful padding of the knees and feet. A 16-gauge needle was used to access the pelvicalyceal system through the targeted calyx under fluoroscopic guidance with injection of contrast material through the ureteric catheter. After introduction of a guidewire through the needle, the percutaneous tract was dilated by serial dilators and established by placing an 18-French access sheath. Nephroscopy was done using a 12-French rigid nephroscope. After stone disintegration using a ballistic lithotripter, the maneuver was concluded by the placement of a 14-French nephrostomy tube that remained in place at least 24 hours postoperatively.

### Postoperative pain management

Starting from admission to the PACU, patients were allowed to be accompanied by one of their parents/care givers. Postoperative pain was scored from 0‒10 based on the Face, Legs, Activity, Cry and Consolability (FLACC) scale.^11^ Pain assessment was done in the PACU at 0.5-, 1-, and 2 hours of arrival; then in the surgical intermediate care unit at 4, 6, 12, and 24 hours. IV paracetamol was prescribed regularly every six hours. If the pain score was ≥ 4 during routine assessment or in between upon patient or relative request, nalbuphine (0.1 mg.kg^-1^) was administered. If the pain score remained ≥ 4, another dose of nalbuphine was administered. Block failure was considered if the patient received two doses of nalbuphine in the PACU.

### Study outcomes

The primary outcome was time to first rescue analgesia (nalbuphine) based on FLACC ≥ 4. The secondary outcomes included the total dose and frequency of nalbuphine in the first 24 hours postoperatively, the time needed to conduct the block, the intraoperative HR and Mean Arterial Pressure (MAP), the maximum inspired sevoflurane concentration, and the incidences of complications.

### Sample size calculation and statistical analysis

Based on a former trial,^5^ in which the time to first rescue analgesia after PVB was 664.4 ± 223.4 minutes, G*Power 3 was used to compute the minimum sample size as 26 patients in each arm to detect an absolute difference of 0.8 in the meantime to first rescue analgesia assuming 40% change and a power of 80%. A two-tailed p-value was considered statistically significant if < 0.05. To compensate for probable dropouts, the sample was increased to 28 patients in each arm.

SPSS-IBM (23.0) was utilized for data analysis. Variables are presented as mean ± standard deviation, number (%), or median (Q1, Q3). Survival analysis with log Rank test and Kaplan-Meier plot was done for time to first rescue analgesia. Numerical variables were compared with independent-samples *t*-test/Mann-Whitney *U* test as appropriate. Chi-Square/Fisher’s exact test was used as appropriate to compare the categorical variables.

## Results

**Figure 1:**
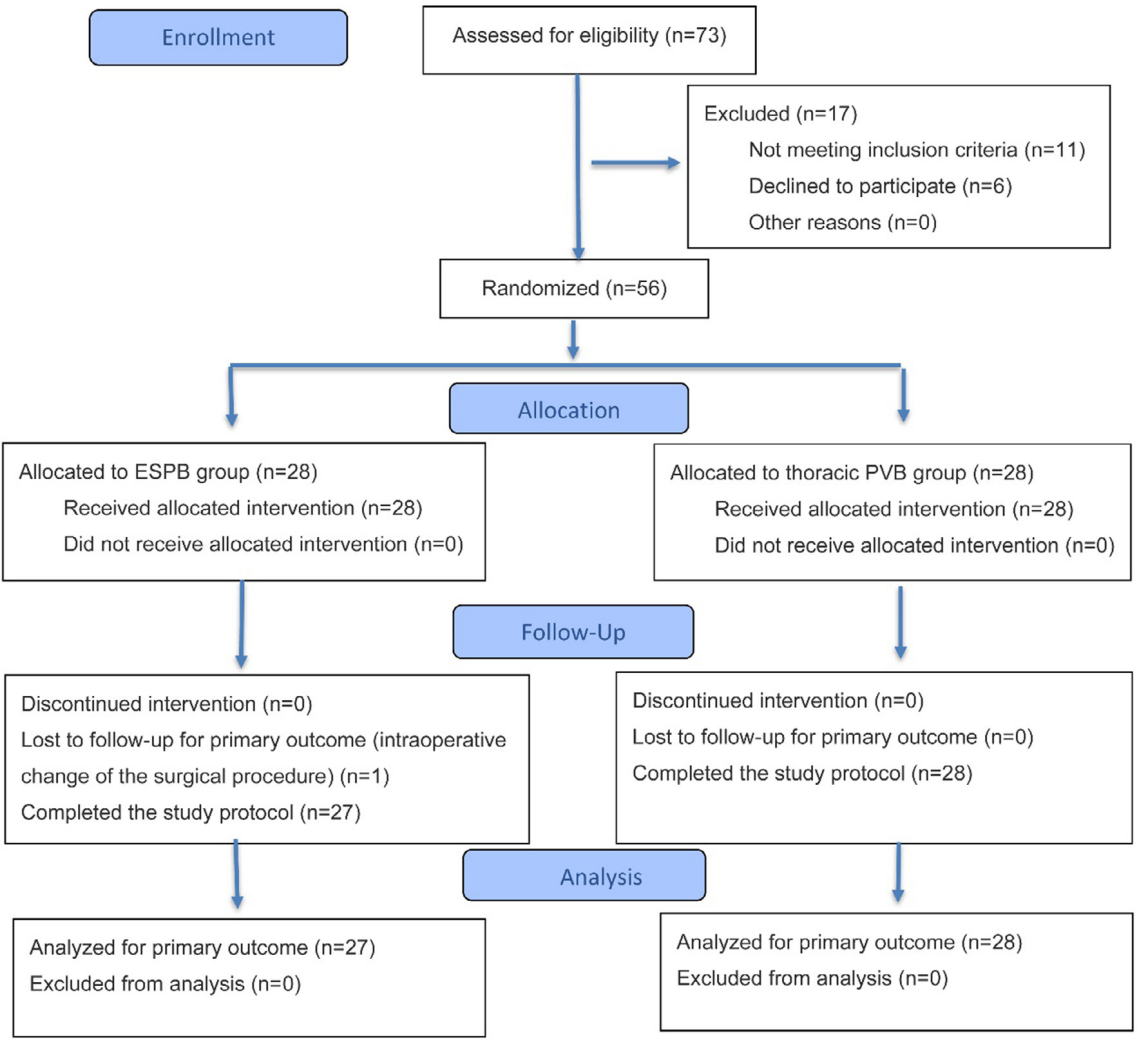
Consolidated standards of reporting trials (CONSORT) flow diagram of the participants. ESPB, Erector Spinae Plane Block; PVB, Paravertebral Block.

From October 2022 to September 2023, we screened 73 patients. Out of them, 56 patients fulfilled the inclusion criteria of this trial and were randomly enrolled into two equal groups to receive ESPB or thoracic PVB. The surgical procedure was changed for one patient intraoperatively. So, the final analysis included 27 patients in the ESPB group and 28 patients in the thoracic PVB group (Fig. 1).

Patients’ characteristics are presented in Table 1. The time to conduct the block was significantly shorter in the ESPB group compared to the thoracic PVB group (p = 0.028). The side and duration of surgery was not significantly different between both groups (Table 1).

**Table 1:** Patients’ characteristics and anesthetic and surgical data.

Parameters	ESPB group (n = 27)	Thoracic PVB group (n = 28)	p-value
Age (years)	4.49 ± 1.66	4.91 ± 1.44	
Gender (male/female)	12/15	11/17	
Weight (kg)	16.0 (13.0‒19.0)	17.0 (15.0‒20.0)	
Height (cm)	106.0 (90.0‒110.0)	107.0 (101‒115)	
ASA PS (I/II)	18/9	15/13	0.322^a^
Time to conduct the block (min)	4.37 ± 1.08	5.05 ± 1.17	0.028^b^
Side of surgery (right/left)	16/11	13/15	0.341^a^
Duration of surgery (min)	86.41 ± 21.89	84.75 ± 31.73	0.823^b^

Data are presented as mean ± SD, median (Q1, Q3), or number.

ESPB, Erector Spinae Plane Block; PVB, Paravertebral Block; ASA PS, American Society of Anesthesiologist Physical Status.

a Chi-Square test.

b Independent sample *t*-test Statistically significant difference is considered as p-value < 0.05.

**Figure 2:**
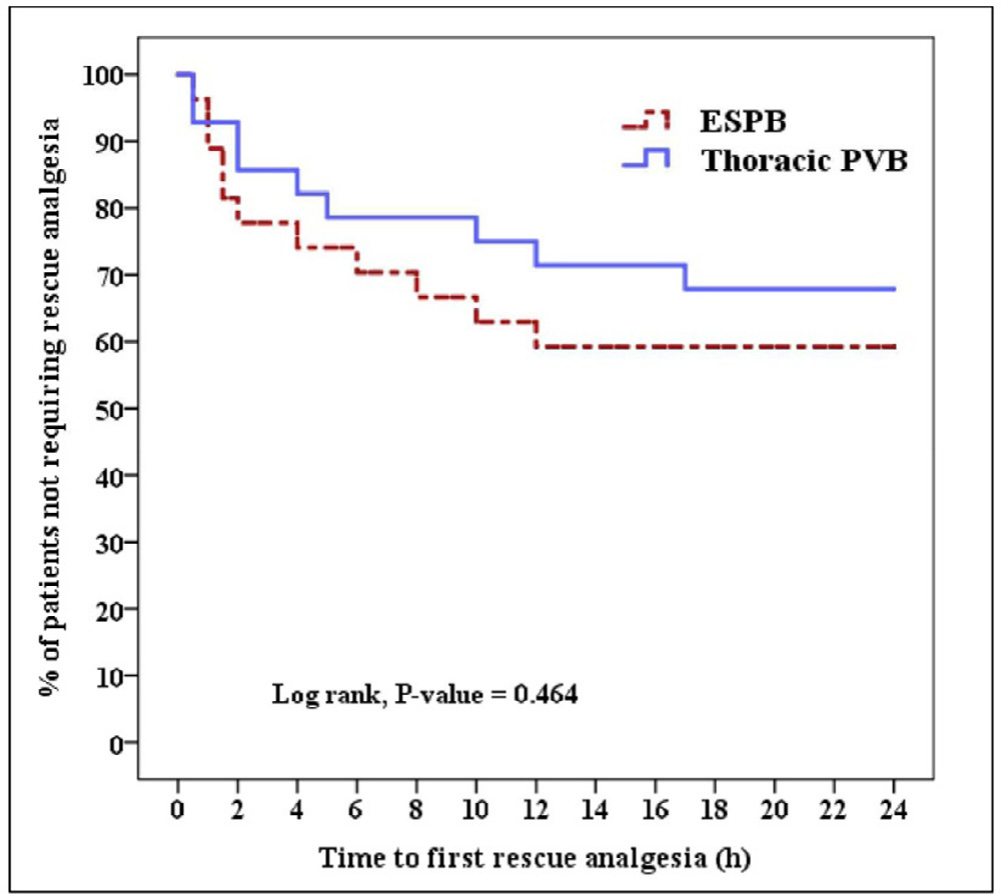
Kaplan-Meier survival plot illustrating the time to first rescue analgesia in both groups. ESPB, Erector Spinae Plane Block; PVB, Paravertebral Block.

The survival analysis and the Kaplan-Meier plot showed no significant difference between both groups in the time to first rescue analgesia postoperatively (15.98 ± 10.17 h [95% CI: 12.14–19.82] in the ESPB group versus 18.18 ± 9.18 h [95% CI: 14.7–21.58] in the thoracic PVB group, [p = 0.464, log-rank test]) (Fig. 2).

The percentages of patients who had FLACC scores < 4 without any rescue analgesia were comparable between both groups during the first 2, 6, 12, and 24 hours postoperatively. Furthermore, during the first 24 hours postoperatively, the total dose and frequency of rescue analgesia showed no significant differences between both groups (Table 2). Only one patient in each group received two doses of nalbuphine in the PACU to achieve a pain score < 4.

**Table 2:** Postoperative pain.

Parameters	ESPB group (n = 27)	Thoracic PVB group (n = 28)	RR (95% CI)	p-value
FLACC score < 4 without any rescue analgesia				
During the first 2 postoperative hours	21 (77.8)	24 (85.7)	0.907 (0.705‒1.167)	0.446^a^
During the first 6 postoperative hours	19 (70.4)	22 (78.6)	0.896 (0.656‒1.224)	0.485^a^
During the first 12 postoperative hours	16 (59.3)	20 (71.4)	0.830 (0.561‒1.226)	0.343^a^
During the first 24 postoperative hours	16 (59.3)	19 (67.9)	0.873 (0.583‒1.307)	0.508^a^
Rescue analgesia during the first 24 hours				
Total dose of nalbuphine (mg)	0 (0, 2)	0 (0, 1.65)		0.488^b^
Frequency of nalbuphine	0 (0, 1)	1 (0, 1)		0.479^b^

Data are presented as number (%) or median (Q1, Q2).

ESPB, Erector Spinae Plane Block; PVB, Paravertebral Block; RR, Relative Risk; CI, Confidence Interval; FLACC, Face, Legs, Activity, Cry, and Consolability.

a Chi-Square test.

b Mann-Whitney *U* test.

Baseline HR and MAP were comparable between both groups, and so were the next intraoperative measurements. Moreover, the percentage of children who had ≥ 20% increase in the HR after nephroscopy did not significantly differ between both groups. There was also no significant difference in the maximum inspired sevoflurane concentration (Table 3). No cases of pneumothorax, Local Anesthetic (LA) toxicity, repeated vomiting, or significant hemodynamic changes were reported.

**Table 3:** Intraoperative data.

Parameters	ESPB group (n = 27)	Thoracic PVB group (n = 28)	95% CI	p-value
Patients with ≥ 20% increase in HR after nephroscopy	1 (3.7)	3 (10.7)		0.319^a^
HR (beats/min)				
Baseline	112.93 ± 19.62	109.57 ± 14.97	-12.77, 6.06	0.478^b^
5 minutes after intubation	113.15 ± 25.33	102.32 ± 13.73	-21.99, 0.37	0.057^b^
Before nephroscopy	100.48 ± 20.03	93.75 ± 10.69	-15.53, 2.07	0.345^b^
1 min after nephroscopy	101.63 ± 20.77	98.71 ± 14.51	-12.58, 6.75	0.893^b^
During skin closure	102.78 ± 19.25	97.21 ± 12.48	-14.31, 3.18	0.427^b^
MAP (mmHg)				
Baseline	71.62 ± 12.10	69.74 ± 17.86	-10.32, 6.57	0.226^b^
5 minutes after intubation	69.7 ± 12.50	64.68 ± 10.11	-11.24, 1.14	0.129^b^
Before nephroscopy	68.59 ± 12.92	71.50 ± 14.39	-4.50, 10.31	0.469^b^
After nephroscopy	70.15 ± 11.61	70.50 ± 11.45	-5.95, 6.65	0.913^b^
During skin closure	75.85 ± 14.67	74.82 ± 12.75	-8.45, 6.39	0.782^b^
Maximum inspired sevoflurane (%)	2.1 (1.8‒2.7)	2 (1.9‒2.4)		0.892^c^

Data are presented as mean ± SD, median (Q1, Q3), or number (%).

ESPB, Erector Spinae Plane Block; PVB, Paravertebral Block; HR, Heart Rate; MAP, Mean Arterial Pressure.

a Fisher’s Exact test.

b Independent sample *t*-test

c Mann-Whitney *U* test.

## Discussion

In this trial, the analgesic efficacy of ESPB was compared to that of thoracic PVB in children undergoing PCNL procedure. The main findings of this trial were that the time to first rescue analgesia and the total dose and frequency of nalbuphine administration in the first 24 hours postoperatively were comparable in both arms.

Over several decades, caudal block was the most common regional block to be conducted with general anesthesia for pediatric patients undergoing infraumbilical surgeries.^12^ However, with the advances in ultrasound technology, new regional techniques emerged with an observed gradual transition from central neuraxial blocks to peripheral and truncal blocks.^13^ An additional advantage of the truncal blocks is the injection of the anesthetic agents in a muscle plane with no need to identify a certain plexus or nerve.^14^ Among those are the thoracic PVB and ESPB.

Providing adequate analgesia for patients undergoing open renal surgeries or PCNL requires blocking both the visceral and somatic innervations to the kidney, ureter, muscle, and skin. This can be readily achieved by ipsilateral paravertebral block at the level of T10‒L1.^15^^,^^16^ In this context, thoracic PVB has been reported in many occasions to provide effective analgesia for open renal surgeries or PCNL procedures in pediatric patients.^4^^,^^5^^,^^17^

Shortly after its first description in 2016,^6^ several case reports and series reported ESPB as part of the analgesia in pediatric patients undergoing numerous surgical procedures.^8^ Holland et al.^18^ reviewed the data of 164 children who received ESPB at a single institution, and reported encouraging results in the surgeries that involved incisions from T1‒L4. Another retrospective observational study reported that ESPB was effective to achieve opioid-free analgesia after different surgical procedures in pediatric patients.^19^ These initial promising findings encouraged the design of Randomized Clinical Trials (RCTs) that reported positive results in pediatric patients undergoing different surgical procedures in terms of reduced postoperative pain scores and decreased intraoperative and postoperative opioid and non-opioid analgesic consumption.^20^, ^21^, ^22^ Furthermore, some trials reported ESPB to be more effective than caudal block in lower abdominal, renal, and hypospadias surgeries.^22^, ^23^, ^24^

In the literature, various studies evaluated using the ESPB for pain management in adult patients undergoing PCNL procedure. Liu et al.,^25^ in a meta-analysis that included 456 adult patients in eight RCTs, described ESPB as a safe and effective technique for this purpose as compared to no block, local anesthetic infiltration, or conventional intravenous analgesia.

To our knowledge, the current trial is the first to compare ESPB with thoracic PVB in pediatric patients undergoing PCNL procedure. Consistent with our findings, Khot et al.^26^ compared both blocks in adult patients undergoing PCNL procedure and reported that both blocks were equally effective in providing postoperative analgesia. Similarly, Fan et al.^27^ compared both blocks for pain management after laparoscopic nephrectomy in adult patients and reported that ESPB provided non-inferior analgesia within the first 24 hours postoperatively.

In PVB, the LA is injected into the Paravertebral Space (PVS) to block the spinal nerves in close proximity to their roots generating ipsilateral sensory, motor, and sympathetic block.^5^^,^^28^ In ESPB, the target is to inject the LA into the erector spinae facial plane, a virtual space beneath the erector spinae muscle that communicates with the PVS. The exact mode of action of ESPB is still not fully understood. However, some imaging and cadaver models showed coverage of the dorsal rami with frequent extension towards the ventral rami and the PVS.^8^^,^^29^ This relative similarity of the mode of action of ESPB and thoracic PVB may explain the comparable analgesic effects of both blocks that are noticed in the current study, and in various previous studies.^26^^,^^27^^,^^30^

In the current study, we used a single-shot technique. This technique has been widely accepted as in both thoracic PVB and ESPB, the injected local anesthetic agents tend to spread caudally and cranially to cover multiple levels.^8^^,^^16^ Moreover, the single-shot technique is less time-consuming and carries less risk of complications. In addition, we found that the time to conduct the block was significantly shorter in the ESPB group. This is consistent with some previous results,^26^^,^^30^ and can be explained by the relative ease to get the sono-anatomic view required to conduct the ESPB. Moreover, the site of injection is superficial compared to the paravertebral space which is deeper and has close proximity to the pleura.^8^^,^^28^ Nevertheless, in the current study, the statistically significant difference in the time to conduct the block between the two studied groups should be interpreted cautiously as it did not reach a clinically important value. Moreover, the current study was not powered for this secondary outcome.

The safety profile of any regional block is a cornerstone that encourages or discourages considering it in clinical practice as part of the anesthetic and analgesic plan. In this study, no incidents of pneumothorax or LA toxicity were mentioned in the two studied groups. In this context, Vecchione et al.,^31^ in their observational study that included 871 pediatric patients, described the thoracic PVB to have low risk of complications. With regard to the ESPB, different studies reported it as associated with no or low risk of complications in the pediatric population undergoing various types of surgeries.^8^^,^^24^ Furthermore, De Cassai et al.^32^ analyzed 45 RCTs that included 1,386 adult patients who received unilateral or bilateral ESPBs for different surgical procedures and reported no complications. Therefore, in general, the ESPB was described to have a better safety profile compared with other loco-regional techniques including the PVB,^29^ and hence it can be advised as an alternative whenever epidural block or PVB are contraindicated or difficult to conduct.

This work had some limitations. First, postoperative pain was assessed by different nurses during the 24-hour period of follow-up. Nevertheless, all the involved nurses were well-trained to use the FLACC scale in pediatric patients. Second, as we assumed a power of 80% during sample size calculation and since no significant difference was detected in the primary outcome, type II error cannot be excluded and small, yet clinically significant, differences might be unrevealed. Third, the sample size might be insufficient to compare the incidence of complications in both blocks.

Finally, we recommend further RCTs with larger sample sizes to confirm the current findings or to detect small, yet clinically significant differences between both groups. We also suggest future RCTs with different volumes/concentrations of the local anesthetic, or with adding adjuvant agents to achieve optimum postoperative pain management.

## Conclusion

ESPB was not found to be more effective than thoracic PVB for postoperative pain management in children undergoing PCNL procedure. Moreover, the intraoperative hemodynamics and maximum sevoflurane concentration were comparable. The time to conduct ESPB was significantly shorter; hence, it could be considered as an easy alternative to thoracic PVB for this procedure.

## Data availability statement

The datasets generated and/or analyzed during the current study are available from the corresponding author upon reasonable request.

## The registry of the study

This study was registered on clinicaltrials.gov

Registration number: NCT05589649

Date of registration: October 18, 2022

## Institutional review board approval

This study was approved from the Institutional Review Board (IRB) of Assiut University Faculty of Medicine, Egypt.

IRB n° 17300787

Date of approval: July 21, 2022

## Authors' contributions

Fatma Nabil: Conceptualization; methodology; writing the original draft.

Ahmed M. Mandour: Methodology; data curation.

Amr M. Abdelgawad: Methodology; investigation.

Deiaaeldin M. Tamer: Methodology; data curation.

Ahmed A. Shahat: Investigation; software; writing the original draft.

Mohamed Anwar: Investigation; writing-review & editing; supervision.

Hany M. Osman: Formal analysis; writing-review & editing; project administration.

## Funding

This research did not receive any specific grant from funding agencies in the public, commercial, or nonprofit sectors.

## Conflicts of interest

The authors declare no conflicts of interest.
